# HLA Class II Histocompatibility Antigen γ Chain (CD74) Expression Is Associated with Immune Cell Infiltration and Favorable Outcome in Breast Cancer

**DOI:** 10.3390/cancers13246179

**Published:** 2021-12-08

**Authors:** Julie B. Noer, Maj-Lis M. Talman, José M. A. Moreira

**Affiliations:** 1 Faculty of Health and Medical Sciences, Institute of Drug Design and Pharmacology, University of Copenhagen, 2100 Copenhagen, Denmark; julie.noer@bio.ku.dk; 2 Diagnostic Center, Department of Pathology, Copenhagen University Hospital, 2100 Copenhagen, Denmark; Maj-Lis.Moeller.Talman@regionh.dk

**Keywords:** breast cancer, cluster of differentiation 74 (CD74), triple-negative breast cancer, immunotherapy

## Abstract

**Simple Summary:**

CD74 is a transmembrane protein normally expressed in immune cells, and aberrantly expressed in cancer cells. Although CD74 overexpression is mostly associated with hematologic malignancies, some studies have also reported CD74 expression in breast cancer especially associated to the triple negative subtype and metastatic breast cancer. The triple-negative breast cancer is generally more aggressive and with a poorer prognosis than the other subtypes. Immunotherapy holds great promise in clinical management of breast cancer, and CD74 may play a regulatory role in immune system responses. Our results showed that CD74 is associated with expression of programmed cell death ligand 1 (PD-L1), which in turn is involved in preventing anticancer immune responses. Overall, our results indicate that CD74 may be a therapeutic target for the treatment of breast cancer patients, in particular in triple negative breast cancer and metastatic breast cancers, where CD74 is commonly overexpressed.

**Abstract:**

The triple-negative breast cancer (TNBC) subtype, defined as negative for ER, PgR, and HER2, is biologically more aggressive and with a poorer prognosis than the other subtypes, in part due to the lack of suitable targeted therapies. Consequently, identification of any potential novel therapeutic option, predictive and/or prognostic biomarker, or any other relevant information that may impact the clinical management of this group of patients is valuable. The HLA class II histocompatibility antigen γ chain, or cluster of differentiation 74 (CD74), has been associated with TNBCs, and poorer survival. However, discordant results have been reported for immunohistochemical studies of CD74 expression in breast cancer. Here we report validation studies for use of a novel CD74 antibody, UMAb231. We used this antibody to stain a TMA including 640 human breast cancer samples, and found no association with the TNBC subtype, but did find a positive correlation with outcome. We also found associations between CD74 expression and immune cell infiltration, and expression of programmed death ligand 1 (PD-L1). Given that CD74 may play a role in innate immune system responses and the potential of immunotherapy as a viable treatment strategy for TNBCs, CD74 expression may have predictive value for immune checkpoint therapies.

## 1. Introduction

Breast cancer (BC) is the most common type of cancer among women worldwide and the leading cause of death from cancer among women [1,2]. BC is not one single disease, but rather a group of intrinsically distinct disease entities or subtypes, such as Luminal A, Luminal B, HER-2 enriched and basal-like (triple negative), which are molecularly heterogeneous and exhibit different prognoses and responses to therapy [3]. Disparate multi-gene expression signatures have been reported that are able to provide prognostic information for BC subtypes. Although there is little overlap in their constituent genes, their predictive value appears to be due mostly to their ability to assess levels of proliferation, and estrogen receptor (ER)- and human epidermal growth factor receptor 2 (HER2)-signaling, three crucial biological processes driving BC [4]. As a result, clinico-pathologic surrogate definitions of intrinsic subtypes, based on immunohistochemical evaluation of ER, progesterone receptor (PgR), HER2, and the Ki67 cell proliferation antigen are broadly accepted as adequate to identify intrinsic subtypes, and guide decisions on clinical management of patients [1,5,6,7,8].

The triple-negative breast cancer (TNBC) subtype expresses none of the three receptors (ER and PgR absent, and HER2 negative, respectively) and is generally more aggressive and with a poorer prognosis than the other subtypes [9,10]. Although no approved targeted therapy is currently available for TNBCs, with cytotoxic chemotherapy remaining the mainstay of treatment for patients with TNBC, extensive molecular characterization has revealed promising molecular targets such as the vascular endothelial growth factor (VEGF), epidermal growth factor receptor (EGFR), and polyadenosine ribose polymerase (PARP) inhibitors [11,12,13]. One particularly promising treatment strategy is immunotherapy. Breast cancers are generally termed “cold tumors” due to a paucity of tumour-infiltrating lymphocytes (TILs) and a generally low mutational burden [14]. However, TNBCs show high mutational loads, and TNBCs are also more likely than any other type of breast cancer to be infiltrated with immune cells, in particular T cells [15]. In addition, in TNBC patients, expression of programmed death ligand 1 (PD-L1) occurs mainly on tumor-infiltrating immune cells rather than on tumor cells, suggesting that disruption of the programmed death 1 (PD-1)/PD-L1 interaction may be a useful treatment strategy for TNBCs [16,17,18,19].

HLA class II histocompatibility antigen γ chain, or cluster of differentiation 74 (CD74), is a trans-membrane protein first discovered as a part of the MHC class II antigen-presenting complex. CD74 has multiple proteoforms [20]. The most common isoform is 33 kDa (p33), but various other isoforms caused by use of an alternative translation initiation site (p35), alternative splicing (p41), or both (p43) also exist. CD74 functions as a chaperone for the correct folding of MHC class II and regulates its antigen-binding capacity [21,22,23]. Furthermore, it acts as a cell-surface receptor for the cytokine macrophage migration inhibitory factor (MIF). MIF binding to CD74′s extracellular domain results in activation of cellular signaling and stimulation of cellular proliferation and survival programs [24,25,26]. The MIF-CD74 axis can also play an important regulatory role in innate immune system responses to cancer by inducing an immunosuppressive environment that supports tumor progression [27,28].

Under physiological conditions, CD74 is expressed together with MHC class II mainly in professional antigen-presenting cells of the immune system. However, under inflammatory conditions, CD74 can be upregulated in certain epithelial cells [29,30]. In cancers, many studies have demonstrated that CD74 is overexpressed in tumor cells. This has been documented in several hematological cancers [31] and in a number of solid tumors, including breast cancer [32]. In breast cancer, CD74 has been described to be overexpressed in both cancer cells and cancer-associated stromal cells [33,34,35,36,37,38,39]. Increased CD74 expression has been correlated with adverse disease parameters, such as triple-negative cancer subtype, increased presence of metastases, and poorer survival, in a number of studies [35,36,37,40]. However, other studies found it to be correlated with favorable parameters such as increased infiltration of the tumor by tumor-infiltrating immune cells and better survival [34,38,41,42]. The presence of infiltrating immune cells in a tumor indicates an activated immune response towards the cancer. Since CD74 expression may mediate both tumor promoting and anti-tumor effects in cancer cells, respectively, the outcome of CD74 overexpression in breast cancers is not easily predictable and may be contingent on multiple parameters. However, given the potential regulatory role that the MIF-CD74 axis may play in innate immune system responses, and the clinical promise of immunotherapy in management of TNBCs, it is essential to clarify some of the conflicting reports on associations between CD74 expression and clinico-pathologic parameters in BC, particularly in TNBCs.

## 2. Materials and Methods

### 2.1. Cell Culturing and Reagents

The human breast cancer cell line MDA-MB-231 was obtained from the American Type Culture Collection (ATCC, Rockville, MD, USA). Cells were grown in Dulbecco’s Modified Eagle Medium (DMEM) + GlutaMAX™ (ThermoFisher Scientific, Waltham, MA, USA) supplemented with 10% fetal bovine serum (FBS) (Sigma-Aldrich, St. Louis, MO, USA) and kept in a humidified atmosphere at 37 °C and 5% CO_2_. Cell line identity was confirmed by short tandem repeat DNA profiling (IdentiCell, Aarhus, Denmark).

### 2.2. CD74 Modulation

CD74 was down-regulated by small interfering ribonucleic acid (siRNA)-mediated gene silencing. An siRNA targeting a common region of the two major human messenger RNA (mRNA) species of CD74 (p31 and p41) was used (sense; 5′-GUCGGAACAGCAGAUAACA[dT][dT]–3′) (Sigma-Aldrich). MISSION^®^ siRNA Universal Negative Control #1 (Sigma-Aldrich) was used as a negative control. Cells were transfected with 25–50 nM of either siRNA using Lipofectamine^®^ 3000 (ThermoFisher Scientific) diluted in Opti-MEM™ (ThermoFisher Scientific) for 4 h, before medium was changed to normal growth medium. For up-regulation of CD74 protein, cells were cultured in the presence of 50 ng/mL recombinant human interferon-γ (IFNγ; ProSpec, Rehovot, Israel) for 48 h.

### 2.3. Protein Extraction and Western Blot Analysis

Total cell lysates were prepared in M-PER™ Mammalian Protein Extraction Reagent containing Pierce Protease and Phosphatase Inhibitor Mini Tablets (ThermoFisher Scientific). Lysate protein concentrations were measured using Pierce™ BCA Protein Assay Kit (ThermoFisher Scientific) and equal protein amounts were mixed with NuPAGE^®^ LDS Sample Buffer (4×) (ThermoFisher Scientific) and DTT and denatured. Samples were loaded and separated by electrophoresis on NuPAGE™ 4–12% Bis-Tris Gels (ThermoFisher Scientific). Proteins were transferred to nitrocellulose membranes using the iBlot™ 2 Gel Transfer Device (ThermoFisher Scientific). Membranes were blocked in 5% (*w*/*v*) skim milk powder (Millipore, MA, USA) or 5% (*w*/*v*) bovine serum albumin (BSA) (Sigma-Aldrich) in tris-buffered saline + 0.05% TWEEN^®^ 20 (TBS-T) for 1 h at room temperature and subsequently incubated overnight at 4 °C with primary antibodies diluted in the appropriate blocking solution. Membranes were washed in TBS-T, incubated with horseradish peroxidase-conjugated secondary antibody (Agilent, Glostrup, Denmark) diluted in blocking solution, washed in TBS-T and then incubated with Clarity™ Western ECL Substrate (Bio-Rad Laboratories, Hercules, CA, USA). Chemiluminescence was detected on UVP BioSpectrum Imaging System. Dynactin/p150^Glued^ was used as a high molecular weight loading control (150 kDa), as other commonly used housekeeping genes, such as actin (53 kDa), β-tubulin (55 kDa), or GAPDH (37 kDa), respectively, all have molecular weights in the same range as CD74 (35 kDa) and CD74 isoforms (p33/35 and p41/43).

### 2.4. Clinical Samples and Tissue Microarray (TMA)

Non-selected formalin-fixed paraffin embedded breast cancer samples (*n* = 37) from a previously described cohort of TNBCs [43] were used for antibody optimization. The project was approved (KF 01-069/03) by the Copenhagen and Frederiksberg regional division of the Danish National Committee on Biomedical Research Ethics. Written informed consent was obtained from each patient included in the study.

To investigate correlations between CD74, PD-L1, and clinicopathologic parameters, a TMA comprising 640 breast cancer samples retrieved from the Yale University Department of Pathology archives (covering patient material collected from 1961 to 1983) was used. The TMA was constructed with single 0.6-mm-diameter tissue cores of each case and has been previously described [44]. The cohort was selected to contain equal numbers of node-positive and node-negative specimens. The mean follow-up time of the cohort is 12.8 years and the mean age of diagnosis is 58.1 years. Most patients received local irradiation, and 15% of lymph node-positive patients received adjuvant chemotherapy. The TMA was provided by the Yale Cancer Center Pathology Tissue Microarray Facility (Table 1).

### 2.5. Immunohistochemistry (IHC)

To allow comparative IHC analysis, cultured cells were washed, trypsinized and then fixed with 4% buffered formalin (Sigma-Aldrich). Following extensive washings with PBS, cells were centrifuged to form a pellet, which was kept in 70% ethanol (Sigma-Aldrich) until embedded in paraffin. Three µm sections cut from FFPE blocks of tissue samples, or cell lines, were mounted onto glass slides. Sections were deparaffinized with xylene and rehydrated in a graded series of ethanol concentrations. Antigen retrieval was performed by heating sections for 10 min in a microwave with Envision Flex Target Retrieval Solution Low pH or High pH (Agilent, Denmark), depending on the antibody. Sections were blocked with 1% H_2_O_2_ for 10 min, permeabilized in TBS + Triton X-100 and incubated overnight at 4 °C with anti-CD74 antibodies (0.4 µg/mL of LN-2 clone or 0.2 µg/mL UMAb231 clone, respectively) diluted in Antibody Diluent (Agilent). The next day, sections were washed in TBS + Triton X-100, incubated with HiDef detection amplifier (Cell Marque, Sigma-Aldrich), washed in TBS + Triton X, incubated with HiDef Detection HRP Polymer Detector (Cell Marque, Sigma-Aldrich), washed and visualized with diaminobenzidine (DAB) chromogen (Agilent). Sections were counterstained with hematoxylin. To allow accurate comparisons, incubation and development times were standardized. Normal rabbit serum instead of primary antibody was generally used as a negative control. Anti-CD74 antibodies were from Sigma-Aldrich (clone LN-2, 1:1500) and from OriGene Technologies (clone UMAb231, 1:2000) and the anti-PD-L1 antibody (clone 28-8, 1:1000) was from Agilent.

### 2.6. Quantitative Assessment of IHC Staining

Slides were scanned using a NanoZoomer-XR Digital slide scanner (Hamamatsu Photonics, Hamamatsu, Japan). Both TMAs and whole slide images were analysed with QuPath digital pathology image analysis software (https://qupath.github.io accessed on 23 January 2020) an open-source imaging platform [45,46]. For TMAs, scanned images were dearrayed within QuPath and all cores were examined prior to scoring to manually exclude those that did not contain tumor cells or were otherwise compromised. Negative, weak, moderate and strong immunostaining thresholds were set manually based upon mean DAB optical densities, and H scores were calculated from the extent and intensity of staining, using a preset algorithm where H-score = 3-fold the % of strongly staining cells + 2-fold the % of moderately staining cells + % of weakly staining cells, giving a score range of 0–300 [47]. 

### 2.7. Data Analysis

Correlations between gene expression of CD74, MIF, CIITA and MHC class II genes and survival (relapse-free survival and overall survival) were analyzed in publicly available breast cancer microarray data using a Kaplan-Meier Plotter online tool (http://kmplot.com/) (accessed on 8 March 2019) [48]. JetSet best probe sets were manually selected for each gene analyzed, if available [49]. Analysis was performed on all subtypes of breast cancer on samples with overall survival (OS) data available. Correlations between CD74, MIF and CIITA mRNA expression with breast cancer tissue infiltration levels of 6 immune cell types (B cells, CD8^+^ T cells, CD4^+^ T cells, macrophages, neutrophils and dendritic cells) were analyzed using the online tool Tumor IMmune Estimation Resource (TIMER) (https://cistrome.shinyapps.io/timer/) (accessed on 8 March 2019) [50,51] from RNA sequencing data obtained from The Cancer Genome Atlas (TCGA).

### 2.8. Statistical Analysis

Pearson’s Chi-squared tests and Wilcoxon rank sum test *p*-values were calculated and plotted in R using the “survplot” function of the “survival” Bioconductor package. Chi-squared test was used to calculate the *p*-values for individual Kaplan-Meier plots.

## 3. Results

### 3.1. Validation and Use of UMAb231, a New Anti-CD74 Antibody

**Figure 1:**
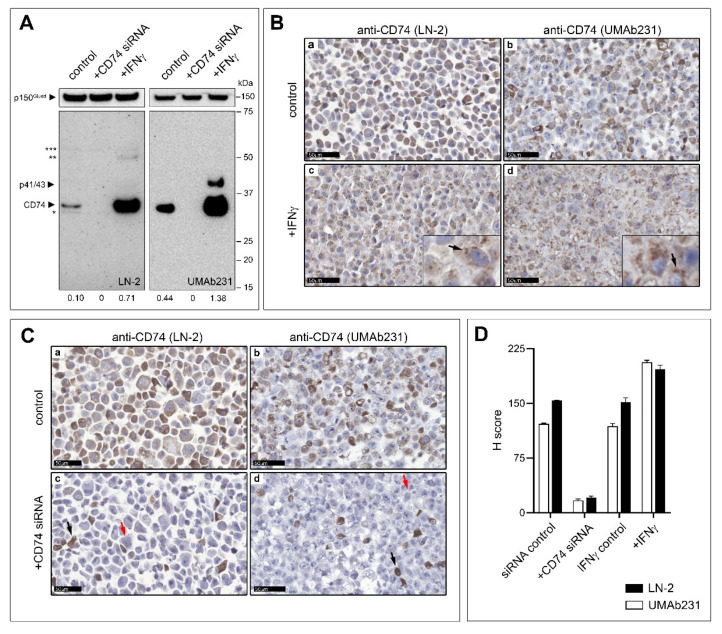
LN-2 and UMAb231 antibody specificity. (**A**) Western blot analysis of CD74 expression was performed by loading whole cell extracts of MDA-MB-231 control cells (control), MDA-MD-231 cells with CD74 siRNA knockdown (+CCD74 siRNA), and IFNγ stimulated MDA-MD-231 cells (+IFNγ). CD74 protein was detected at 33/35 kDa using either anti-CD74 LN-2 (left-hand panel) or UMAb231 monoclonal antibody (right-hand panel), and at 41/43 kDa with UMAb231 in overexpressing cells. *, **, and *** mark the positions of three independent bands of unknown identity. No signal was detected in siRNA-mediated CD74 knockdown cells (+CD74 siRNA). IHC analysis of FFPE sections of MDA-MB-231 breast cancer control cells compared to (**B**) CD74 siRNA knockdown, and (**C**) IFNγ stimulation showed similar staining patterns with LN-2 and UMAb231 antibodies. Insets in subpanels 1b and 1c show higher magnification images illustrating punctuated stainings of CD74. (**D**) Quantification of stainings showed that IHC immunoreactivity correlated with expression of cellular CD74 protein. Scale bars = 20 µm. The uncropped blot is shown in Supplementary material Figure S1.

Many of the studies addressing the effect of CD74 in breast cancer have relied on immunohistochemistry (IHC) with an anti-CD74 mouse monoclonal antibody (LN-2) [52,53]. The conflicting observations reported in the literature could be due to inherent technical issues, such as cross-reactivity due to off-target binding of antibodies, or differential recognition of the various proteoforms of CD74 [54,55]. Recently, a new anti-CD74 mouse monoclonal antibody, UMAb231, was developed by Origene Technologies (Rockville, MD, USA). This antibody was tested for specificity on a high-density 17K protein microarray chip containing 16,242 overexpression lysates, and displayed one single positive reactive protein, CD74, thus demonstrating remarkable specificity under the conditions of the assay. We validated the specificity of UMAb231 for IHC using the human breast cancer cell line MDA-MB-231. This cell line has a robust endogenous expression of CD74 protein, which can be easily upregulated with IFNγ or suppressed with siRNA [35,39,56]. Western blot analysis of whole cells extracts with endogenous expression (control lanes; Figure 1A), siRNA-mediated depletion- (+CD74 siRNA lanes; Figure 1A) or IFNγ-mediated up-regulation (+IFNγ lanes; Figure 1A) of CD74, showed that the LN-2 antibody recognized a major band at 35 kDa, congruent with the expected molecular weight for CD74. A minor band of 33 kDa (labelled with *; Figure 1A) was also seen. Both bands disappeared when CD74 was knocked-down with siRNA (+CD74 siRNA; Figure 1A left-hand panel). The UMAb231 antibody recognized a single band at 35kDa (control; Figure 1A right hand panel), compatible with CD74. This band disappeared in the cells with siRNA-mediated knockdown of CD74 and became much more prominent in IFNγ stimulated cells, respectively (Figure 1A; right hand panel, +CD74 siRNA and +IFNγ, respectively). In IFNγ stimulated cells, an additional band of approximately 41 kDa, compatible with the CD74 p41/p43 isoforms, was also detected with UMAb231 (labelled p41/43; Figure 1A, +IFNγ right hand panel). For LN-2, IFNγ stimulation resulted, in addition to a stronger band for CD74, in the detection of an additional band of approximately 50 kDa (labelled **; Figure 1A). Moreover, a weak unspecific 55 kDa band was also observed with LN-2 under all conditions tested (labelled ***; Figure 1A). These data showed that both LN-2 and UMAb231 recognized CD74 in western blot assays, but showed different specificity patterns.

To validate use of UMAb231 for immunohistochemical analysis of tissue samples, we formalin fixed and paraffin embedded MDA-MB-231 cells, mimicking tissue processing conditions. Sections were stained by IHC with the UMAb231 antibody. MDA-MB-231 cells showed moderate, predominantly cytoplasmic, heterogeneous CD74 staining under endogenous conditions (Figure 1B, panel b). Upon exposure to IFNγ, cellular expression of CD74 became homogenously strong and with a marked presence in cytoplasmic granules (Figure 1B; panel d inset, black arrow). In cells where we knocked-down CD74 with siRNA, we observed complete lack of immunoreactivity for CD74 in >80% of the cells (Figure 1C; panel d, red arrow), with only a few cells presenting CD74 staining (Figure 1C; panel d, black arrow) at levels comparable to those of the control cells (Figure 1C; panel b). For comparative purposes we stained serial sections with anti-CD74 antibody (LN-2) and obtained similar results to those of UMAb231 (compare Figure 1B,C; panels a with b, and c with d, respectively). H-scores generated by image quantification with the QuPath software, verified the similarity in immunostaining results for both antibodies (Figure 1D).

**Figure 2:**
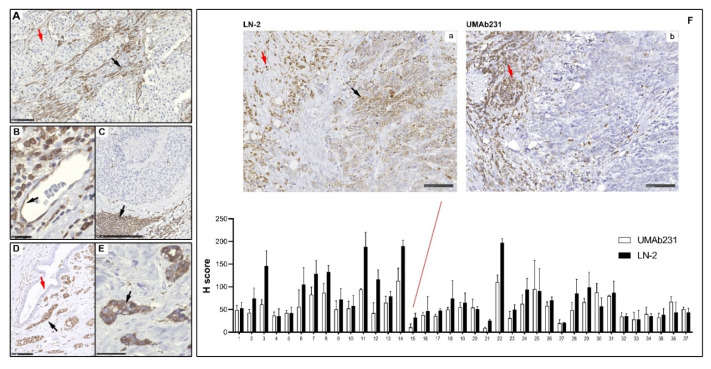
IHC staining patterns of UMAb231. Immunoreactivity for CD74 was seen primarily in stromal components, (**A**) in peritumoral fibroblasts, (**B**) endothelial cells, and (**C**) lymphocytes and macrophages, respectively (black arrows). Staining of serial sections with vimentin, CD4, CD8, CD68 or CD31 was used to confirm cell identity. In some samples, CD74 was also expressed in (**D**) tumor cells (black arrow indicates tumor cells), with essentially cytoplasmic and (**E**) membrane staining (black arrow indicates membrane staining). (**F**) Comparative semi-quantitative analysis of the UMAb231 and LN-2 antibodies, showed similar, but not always identical, patterns between the two antibodies. A case is highlighted with no CD74 immunoreactivity in tumor cells with UMAb231 (sub-panel b), but weak to moderate reactivity with LN-2 (sub-panel a, black arrow). In both cases lymphocytes and macrophages stained strongly with either antibody (sub-panels a and b, red arrows, respectively). Scale bars are 100 µm in (**A**,**D**,**F**), 25 µm in B, 250 µm in (**C**), and 50 µm in (**E**), respectively.

Having established the specificity of UMAb231 for IHC, we proceeded to analyze a set of breast cancer samples. We have previously reported on the proteomic profiling of a cohort of TNBCs [43]. Of these, 37 samples had enough remaining tumor tissue to allow us to determine IHC staining patterns for UMAb231 (Figure 2). As expected, we found reactivity for CD74 in both tumor cells and in stromal components. In the latter, peritumoral fibroblasts (Figure 2A, black arrow), and endothelial cells, as well as lymphocytes and macrophages showed immunostaining for CD74 (Figure 2B,C, respectively). Twelve of the samples showed marked overexpression of CD74 in tumor cells (Figure 2D, black arrow indicates tumor cells), with essentially cytoplasmic and membrane staining (Figure 2E, black arrow indicates membrane staining). Although the staining patterns obtained with the UMAb231 antibody were mostly analogous to those of the LN-2 antibody (Figure 2F), a comparative analysis of the LN-2 and UMAb231 antibodies showed they were not identical. In three cases (Figure 2F, #15, #21 and #27), we found no CD74 immunoreactivity in tumor cells with UMAb231 (Figure 2F; panel b), but weak to moderate reactivity with LN-2 (Figure 2F; panel a, black arrow). In both cases lymphocytes and macrophages stained strongly with either antibody (Figure 2F, panels a and b, red arrows, respectively).

### 3.2. Tissue Microarray (TMA) Analysis of CD74 Expression in BC

**Figure 3:**
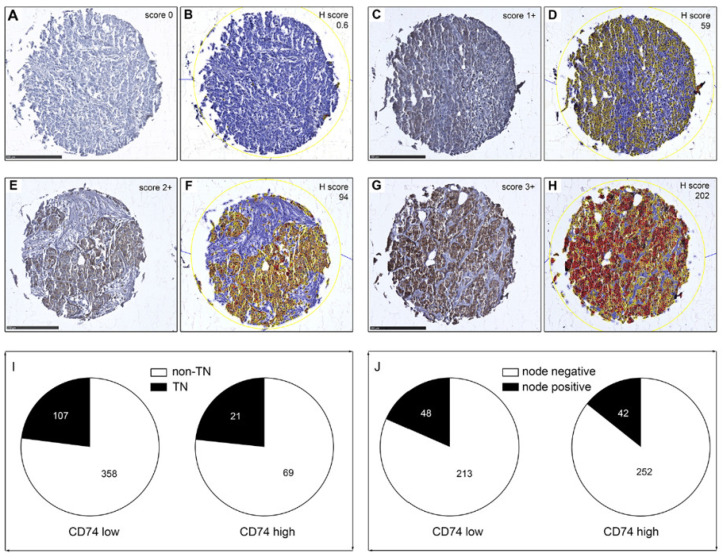
IHC analysis of YTMA49. TMA cores were scored in a categorical manner according to CD74 immunoreactivity levels in tumor cells only, as follows: 0 (absent), 1+ (weak), 2+ (moderate) or 3+ (strong) ((**A**,**C**,**E**,**G**), respectively). An H-score value was also derived by computer-assisted whole tissue core analysis (**B**,**D**,**F**,**H**). Negative, weak, moderate and strong immunostaining thresholds are yellow, orange, and red, respectively. Intensity scores were correlated to (**I**) TN status and (**J**) node status. Scale bars = 250 µm.

Having established the specificity of UMAb231, we stained a TMA including 640 human breast cancer samples (YTMA49) with this antibody. YTMA49 comprises a selected cohort of invasive ductal carcinomas from the Yale University Department of Pathology archives (dated 1961 to 1983), and contains approximately half ER-positive and half ER-negative specimens [57]. The TMA is also balanced in terms of node-positivity (specimens and half node-negative specimens. A total of 555 cores (87% core representation) were present, contained tumor cells, and could be scored reliably. Clinico-pathological parameters are presented in Table 1. Immunostaining of CD74 was scored in two different ways: only considering immunoreactivity in tumor cells or in the entirety of the tissue section (with an H-score). Stainings were scored either in a categorical manner according to CD74 immunoreactivity levels in tumor cells only, as follows: 0 (absent), 1+ (weak), 2+ (moderate) or 3+ (strong) (illustrated in Figure 3A,C,E,G, respectively). Or deriving an H-score value that included whole tissue core analysis using QuPath Software (illustrated in Figure 3B,D,F,H). We found that CD74 was expressed at any level in tumor cells in 164 of the 555 cores (1+, 2+, and 3+ scores; 29.5%); CD74 was also expressed in immune cells in more than half of the samples (342 out of 555 samples, or 61.6%), which was consistent with its known physiological expression in cells such as dendritic cells and B cells [58].

Previous studies have found CD74 expression in human breast cancers to be correlated with generally adverse disease parameters such as increased presence of metastases and triple-negative status of the breast cancer [35,36,37]. In our cohort, the clinical data included the pathology scores for ER, PR and HER2, which allowed us to identify the triple-negative cases (defined as negative for all three receptors). Moderate to high CD74 expression (defined as a CD74 intensity score of 2+ or 3+) was not significantly correlated with the triple-negative subtype compared the cases with absent to low CD74 expression (CD74 intensity score of 0 or 1+) (Table 2, Figure 3I). We defined prevalence of lymph node metastases as the proportion of patients with at least one positive regional lymph node metastasis at diagnosis. CD74 status was not significantly correlated to cancer cell-positive lymph nodes (Table 2, Figure 3J). In addition, CD74 expression was not significantly correlated with patient age, tumor size, ER, PR or HER2 status (Table 2).

**Figure 4:**
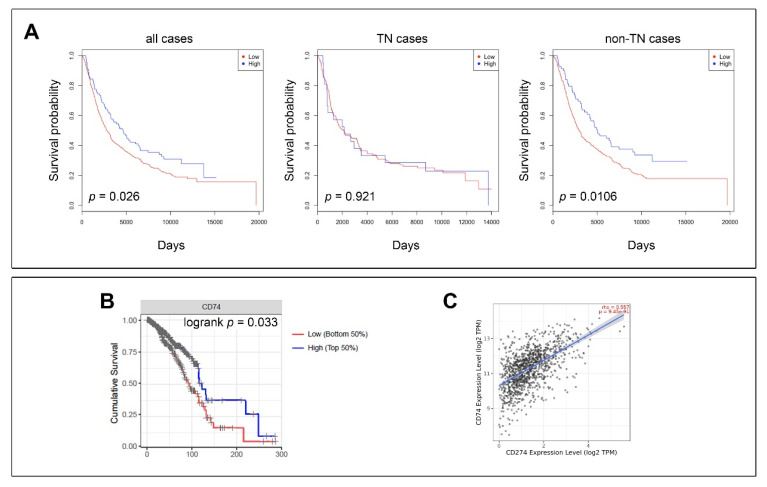
Survival analysis of breast cancer samples as a function of CD74 expression. (**A**) The prognostic value of CD74 expression was examined in the YTMA49 cohort for all cases, TN cases, and non-TN cases. (**B**) Kaplan–Meier plot showing OS among breast cancer patients according to CD74 expression in a multivariate model correcting for immune cell infiltration in a multivariable Cox proportional hazard model. Correlation between *CD74* gene expression and infiltration of immune cells was examined in the breast cancer samples available from TCGA database with the TIMER analysis tool (http://timer.cistrome.org) (accessed on 8 March 2019) (**C**) Correlation between CD74 and PD-L1 in 1017 breast carcinomas from TCGA after correcting for tumor-infiltrating immune cells.

Kaplan-Meier analysis of the overall survival (OS) using CD74 expression scores as categorical variables, comparing patients with low-expression CD74 tumors to those with high CD74 expression, showed that the CD74 high group had significantly better OS (*p* = 0.026; Figure 4A, all cases). There was no difference in OS between CD74 low- and high-expression cancers in the TN subset of this cohort (Figure 4A, TN cases). On the contrary, the OS difference was greater in the non-TN cases, as a whole (*p* = 0.0106; Figure 4A, non-TN cases). Survival analysis using the H-scores calculated with QuPath Software as a continuous variable, showed no significant difference in survival with respect to CD74 expression. Median value and upper and lower quartiles (Q1 vs. Q4) were also used as cut-points for binary split of the H-score. In all cases, there was no significant difference between the two groups.

To confirm the prognostic value of CD74, we used an online integrative survival analysis tool that collects gene expression data and survival information of 1809 breast cancer patients (http://kmplot.com) (accessed on 8 March 2019) [48]. Cohorts were divided into two groups according to the median expression of CD74, and the two groups compared in terms of OS, relapse free survival (RFS) and distant metastasis free survival (DMFS). In all cases the high CD74-expressing patient group fared significantly better than those patients bearing tumors with lower CD74 expression, measured by OS [HR = 0.61 (0.49–0.76)], RFS [HR = 0.70 (0.63–0.78)], or DMFS [HR = 0.82 (0.67–0.99)]. This trend towards a better outcome for patients with highest CD74 expression was seen for all intrinsic subtypes (luminal A, luminal B, HER-2 enriched, and basal-like), but the greatest difference in outcome between the CD74 low and high expressing groups was observed for the basal-like subtype, with RFS [HR = 0.44 (0.34–0.57)]. 

### 3.3. Immune Cell Infiltration and CD74 Expression

IFNγ is known to stimulate upregulation of CD74 protein in many cell types, including in cancer cells (Figure 1) [35,59,60]. As IFNγ is secreted by macrophages and CD4^+^ and CD8^+^ T lymphocytes in the tumor microenvironment, it was conceivable that CD74 overexpression in solid tumors, and prognostic value, is simply a reflection of the level of immune infiltration [61,62]. Using the Tumor IMmune Estimation Resource (TIMER) [50] we examined the correlation between CD74 mRNA expression and infiltration of immune cells in the 1017 breast carcinomas available from The Cancer Genome Atlas (TCGA) available through the TIMER analysis tool (http://timer.cistrome.org) (accessed on 8 March 2019). In this dataset, CD74 expression was negatively correlated with the purity of the samples (Spearman’s ρ = −0.466, *p* < 0.05), and significantly correlated with the presence of all six tested immune cell types, namely B cells (Spearman’s ρ = 0.486, *p* < 0.05), CD4^+^ (Spearman’s ρ = −0.490, *p* < 0.05) and CD8^+^ (Spearman’s ρ = 0.320, *p* < 0.05) T cells, macrophages (Spearman’s ρ = 0.215, *p* < 0.05), neutrophils (Spearman’s ρ = −0.534, *p* < 0.05) and dendritic cells (Spearman’s ρ = 0.633, *p* < 0.05). Survival analysis using a multivariate Cox proportional hazards model on this breast cancer cohort (model: B_cell + CD8_Tcell + CD4_Tcell + Macrophage + Neutrophil + Dendritic + CD74), showed that from the six tested immune cell types and CD74 expression (dichotomized by median into low/high), only CD74 expression had independent prognostic value (Figure 4B; logrank *p* = 0.033).

CD74 is essential for folding and stabilization of HLA class II molecules, suggesting a key role for CD74 in the functional HLA class II processing machinery [55]. The association we observed between CD74 expression and immune cell infiltration, as well as the correlation with outcome, indicated a possible link to immune responses. Given the importance of a functioning antigen-processing machinery for a successful immunotherapy response, and the potential of immunotherapy as a viable treatment strategy for TNBCs, we examined whether expression of programmed death ligand 1 (PD-L1) was in any way associated to CD74 expression. We stained the breast cancer YTMA49 array with an anti-PD-L1 antibody [clone 28-8] and, as before, derived an H-score value for all stainings by whole tissue core analysis using QuPath Software. Correlation of H-scores showed that PD-L1 expression was significantly associated with CD74 expression (Spearman’s ρ = 0.3356; *p* = 0.04). Expression of CD74 was also significantly correlated with PD-L1 expression in the 1017 breast carcinomas available from the TIMER online analysis tool, even after correcting for tumor-infiltrating immune cells (Figure 4C; *p* < 0.001).

## 4. Discussion

Previous studies examining CD74 expression in relation to clinical parameters in breast cancer have come to conflicting conclusions. Some studies found it to be correlated with the presence of metastases, worse outcome, and receptor status (TNBC subtype), whereas other studies found CD74 expression to be correlated with longer survival and immune infiltration [33,38,41,42,63]. Similar discordant findings have been published for other cancers, including melanoma, malignant pleural mesothelioma and brain metastases of several cancer types [64]. Some studies have examined CD74 protein expression by mass spectrometry, or IHC using various antibodies; others investigated CD74 gene expression by microarray or RNA-seq. Moreover, patient cohort sizes in these studies have also varied widely, from as few as 12 to as many as 588 patients. We have validated a novel anti-CD74 antibody, UMAb231, for specificity in detection of the CD74 protein by IHC in human FFPE tissue. Although similar in performance to the commonly used anti-CD74 LN-2 antibody, we found that the UMAb231 antibody showed better dynamic range in the discrimination between endogenous and IFN-γ upregulated levels. Correlation of high CD74 expression in 555 breast cancer samples from a TMA stained with UMAb231 samples and patient clinical data indicated that high expression of CD74 protein was positively associated with OS. This was further validated by examining the correlation between CD74 mRNA expression and OS in a public microarray dataset from 1402 patients. Using the online resource TIMER, we also found results that support that CD74 mRNA expression in breast cancer was associated with increased infiltration by immune cells.

Our results are consistent with the group of previous studies that showed a survival benefit in cancer patients with high CD74 expression and higher immune infiltration [64,65,66]. Unlike previously reported results, we found no correlation between CD74 and the presence of metastases or triple-negative status. In a 2016 study by Wang and colleagues, the survival benefit associated with high CD74 expression was apparently confined to the basal-like subset of the patients [38]. In our own results, the opposite was the case, with the caveat that we looked at the triple-negative subtype, however this subtype overlaps with basal-like lesions to a large extent [9]. When we compared the survival in public microarray data according to luminal A, luminal B, HER2 and basal-like subtypes, we saw a trend more similar to the results reported by Wang and colleagues. However, the difference in survival between low and high CD74-expressing subsets was not clearly confined to the basal-like subset. Instead, the separation of the curves and the significance level seemed to be graded according to the subtypes level of immunogenicity as described in the literature previously (basal-like and HER2 > luminal B > luminal A) [67]. This could be speculated to reflect one of two scenarios: CD74 and its antigen presentation-related functions requires the presence of an immune infiltrate to have an anti-cancer effect. Alternatively, that more immune cell-infiltrated cancers have more of their CD74 mRNA contributed by immune cells and that this results in a larger survival difference, rather than the CD74 expressed endogenously in cancer cells.

To address which of the functions of CD74 is more likely to be associated with survival benefit and immune infiltration we ran the same analysis in the public databases for MIF, CIITA and different MHC class II genes. Based on this, CD74’s correlation with survival and immune infiltration are more likely to be associated with the MHC class II function. Studies on the molecular functions of CD74 in cancer cells have generally found that the MIF-CD74 signaling pathway stimulates processes that would support cancer progression such as increased cancer cell proliferation, survival and invasion [68,69]. However, almost all pre-clinical studies on CD74 in cancer are conducted in vitro or, in a few cases, in immunodeficient mouse models. Thus, the known link between CD74 and the immune system has not been considered. It is thoroughly established that CD74 has regulatory functions for antigen presentation by MHC class II. Antigen-presentation is important for anti-tumor immune responses. However, this has mostly been attributed to MHC class I. Zeiner and colleagues recently published a study showing that knockdown of CD74 in brain-seeking melanoma cells decreased the complexity of the MHC class II peptidome. CD74 tumor cell expression in clinical samples of brain metastases was here also associated with better overall survival, which indicates that indeed the MHC class II-related functions of CD74 are also important in the context of cancer.

The correlations between CD74 protein or mRNA levels and survival from both the TMA and microarray results must be interpreted with some caution. We can conclude from the staining of whole slide sections of larger tumor specimens that CD74 appears to be heterogeneously expressed in different parts of a tumor, and the smaller biopsy cores from a TMA may not always represent the greater whole of a tumor. Furthermore, at the mRNA level in homogenized tissue samples, it is likely that infiltrating immune cells contribute some of the CD74 mRNA measured. Nonetheless, the same overall trend of longer OS in CD74 high-expressing patients was evident both at the protein level in a TMA of 555 samples and a gene expression dataset of 1402 samples. The results from public microarrays had a larger and more significant survival difference than the TMA results, which could be explained by some of the CD74 mRNA coming from immune cells. It should be noted that when survival in the TMA cohort was compared according to automatically calculated H scores, there was no detectable difference in survival. This readout should be more comparable to gene expression-based results, as it measures all staining regardless of cellular origin. Thus, this indicates that there is certainly cohort- and readout-based uncertainties in determining the clinical significance of CD74 expression in cancer cells and associated immune cells. The results from the TIMER resource also indicated that CD74 expression was inversely correlated with tumor purity. The partial correlations with immune cell infiltration from TIMER were corrected for sample purity, but due to the likely contribution of immune cells to the CD74 mRNA pool conclusions should also be drawn from these with caution. The correlation with the presence of dendritic cells and neutrophils were the highest (*p* = 1.03 × 10^−107^ and *p* = 4.36 × 10^−71^, respectively). CD74 is known to be mainly expressed in B cells and dendritic cells, but also to some extent in macrophages, monocytes and subsets of activated T cells [31]. Since the purity corrected *p*-values were still highly significant for CD74, it is indicated that CD74 is marker of immune infiltration in breast cancer, but from these results, it is hard to pinpoint a certain immune cell type it is associated with.

Finally, we also found associations between CD74 expression and expression of programmed death ligand 1 (PD-L1). Given that expression of both CD74 and PD-L1 can be induced by proinflammatory cytokines such as IFNγ, it is possible that the association we found simply reflects the presence of cytokines produced by activated CD8^+^ T cells in the tumor microenvironment, rather than a functional relationship. However, since CD74 expression may impact anti-tumor immune responses, decreased MHC class II expression may lead to reduced tumor cell immunogenicity.

## 5. Conclusions

We validated a novel anti-CD74 antibody, UMAb231, for usage in immunohistochemical assays. Analysis of human breast cancer samples showed no association with the TNBC subtype, but did find a positive correlation with outcome. We also found associations between CD74 expression and immune cell infiltration, and CD74 expression and PD-L1 expression. More research is needed to determine whether the survival benefit and immune infiltration associated with CD74 and MHC class II are linked to the function of CD74 in MHC class II-mediated antigen presentation, or it mainly comes from MHC class IIs own functions and the correlation with CD74 is merely a consequence of their commonly regulated expression. Our results support the hypothesis that CD74 expressed in breast cancer is a marker of immune cell infiltration and longer survival.

## Figures and Tables

**Table 1:** Patient characteristics in YTMA-49.

Parameter	N	Mean/Percentage of Cases (%)
Age (mean)	555	58.0 years
Lymph node status		
Negative	261	47%
Positive	294	53%
ER status		
Negative	270	49%
Positive	285	51%
PR status		
Negative	294	53%
Positive	261	47%
HER2 status		
Negative	359	65%
Positive	196	35%
TN status		
Non-TN	427	77%
TN	128	23%

**Table 2:** Low/high score association with pathology scores, nodal status and TN status (categorical variables).

	CD74 Low	CD74 High	*p*-Value
Age (years)	58.0 (56.9–59.2)	57.7 (55.2–60.2)	0.7601
ER status			0.8567
Negative	227	43	
Positive	238	47	
PR status			0.1235
Negative	253	41	
Positive	212	49	
HER2 status			0.9585
Negative	301	58	
Positive	164	32	
Nodal status			0.1904
Negative	213	48	
Positive	252	42	
TN status			0.947
Not TN	358	69	
TN	107	21	

## Supplementary Materials

The following are available online at https://www.mdpi.com/article/10.3390/cancers13246179/s1, Figure S1: The uncropped blot is shown in Supplementary material.
