# Supplementary figures and images for: HLA Class II Histocompatibility Antigen γ Chain (CD74) Expression Is Associated with Immune Cell Infiltration and Favorable Outcome in Breast Cancer
Source: Cancers (Basel). 2021 Dec 8;13(24):6179. doi: 10.3390/cancers13246179 (PMC8699420; doi:10.3390/cancers13246179)

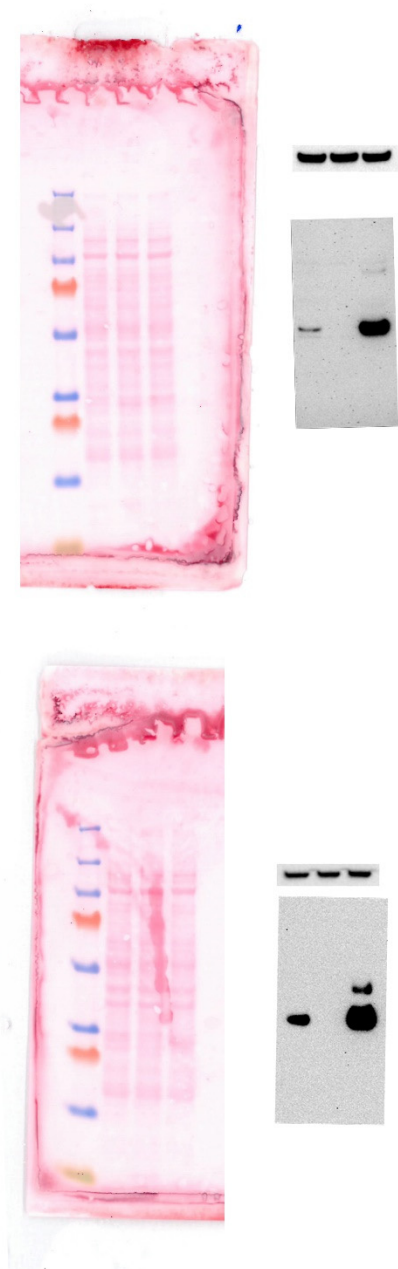

**Figure S1.** The uncropped blot.

Supplement: Supplementary file 1 [file cancers-13-06179-s001.zip › cancers-1408718-supplementary.pdf]
